# Simulation-Omitting and Using Library Patients for Pre-Planning Online Adaptive Radiotherapy (SUPPORT): A Feasibility Study for Spine Stereotactic Ablative Radiotherapy (SAbR) Patients

**DOI:** 10.3390/cancers17071216

**Published:** 2025-04-03

**Authors:** Da Wang, Heejung Kim, Tingliang Zhuang, Justin D. Visak, Bin Cai, David D. M. Parsons, Steve Jiang, Andrew R. Godley, Mu-Han Lin

**Affiliations:** Department of Radiation Oncology, University of Texas Southwestern Medical Center, Dallas, TX 75309, USA; da.wang@utsouthwestern.edu (D.W.); heejung.kim@utsouthwestern.edu (H.K.); tingliang.zhuang@utsouthwestern.edu (T.Z.); justin.visak@utsouthwestern.edu (J.D.V.); bin.cai@utsouthwestern.edu (B.C.); david.parsons@utsouthwestern.edu (D.D.M.P.); steve.jiang@utsouthwestern.edu (S.J.); andrew.godley@utsouthwestern.edu (A.R.G.)

**Keywords:** adaptive radiotherapy, simulation free, planning free, spine SAbR, inter-patient ART, robust planning, Hypersight CBCT

## Abstract

Purpose: Spine treatment can be emergent, and it is desirable to expedite the process. This study aims to leverage the online adaptive radiotherapy (ART) system to develop and validate simulation-omitted spine stereotactic ablative radiotherapy (SAbR) using X-ray-based ART. This could potentially eliminate the necessity for patient-specific simulation computed tomography (CT) and conventional treatment planning. Methods: To demonstrate the feasibility of the SUPPORT treatment strategy, we used a pre-clinical release version of the Ethos Emulator 2.0 treatment planning system and ART emulator. In our study, the prescriptions were 14 Gy in one fraction to the vertebral body with a simultaneous integrated boost to 20 Gy. The simulation CTs of nine patients were selected to create a library patient pool. They were used for pre-planning purposes. Another five thoracic spine (T spine) and five lumbar spine (L spine) test patients with Hypersight cone beam computed tomography (CBCT) were selected for online ART in this study. Ethos emulator 2.0 was used to generate an online adaptive plan on the test patients’ Hypersight CBCT using the pre-plans from the library patients. The planning target volume (PTV) coverage and organ-at-risk (OAR) doses were evaluated. Results: All ten test patients met PTV coverage above 90% of the PTVs and had no violation of the spinal cord or other OAR dose constraints. The online adaptive plans and pre-plans have a similar quality. The average difference in the coverage of PTV 20 Gy and PTV 14 Gy is 1.05% (0.1–3.4%) and 1.76% (0.2–6.8%), respectively. The average difference in spinal cord dosimetry is 0.67 Gy (0.09–2.5 Gy) for maximum dose and 0.09cc (0–0.61cc) for V10 Gy. Conclusion: This study lays the groundwork for future investigations on the feasibility of treating spine SAbR patients without the need for simulation CT. The online adaptive plans generated through our workflow achieved a quality similar to the pre-plans of the library patients.

## Simple Summary

This standard radiation therapy workflow requires CT simulation and treatment planning before initiating treatments, which typically takes one to two weeks. This study explores a new approach to treating spine SAbR patients more quickly by eliminating CT scans and planning. Instead, we use existing plans from previous patients and adapt them to new patients using a technology called online ART. Patients receive the right amount of radiation to the tumor while avoiding damage to the spinal cord. This approach significantly speeds up the process, allowing patients to start treatment much faster, especially in urgent cases. Overall, it makes the treatment process more efficient and can improve care for patients who need immediate attention.

## 1. Introduction

Treatment planning in the field of radiation therapy has evolved from three-dimensional (3D) planning to inverse planning and, most recently, to personalized adaptive radiotherapy (ART) [1]. These technical advancements enable enhanced target conformity and improved sparing of organs at risk (OARs), even at the inter-fraction level [2]. Traditional inverse planning techniques, such as intensity-modulated radiotherapy (IMRT) [3] and volumetric modulated arc therapy (VMAT) [4,5], typically require extended planning time and a comprehensive quality assurance (QA) process compared to 3D planning [6]. After patients’ CT simulations, it often takes one to two weeks for the treatment plans to be finalized and ready for safe delivery [7,8]. However, this lengthy process may delay urgent patients’ access to radiation therapy. In such cases, physicians may rely on a 3D approach [9,10,11]. For other patients who can tolerate a delayed treatment start, this delay may still negatively impact tumor control and overall treatment outcomes [12,13,14,15,16,17]. Therefore, it is important to reduce the waiting time to improve patients’ outcomes in radiation therapy.

Recently, simulation CT-omitted (sim-omitted) radiotherapy has been explored to utilize diagnostic images for planning purposes with the aim of reducing patients’ wait time [18,19,20,21,22,23,24]. In sim-omitted treatments, the planning process can begin following the acquisition of diagnostic images rather than traditional simulation CT. This approach allows patients to receive treatment on the same day as the consultation in radiation oncology. For sim-omitted 3D treatments, studies have demonstrated that the uncertainties associated with planning on diagnostic images are generally within acceptable limits [24]. The clinical outcomes of sim-omitted 3D treatments are being investigated in an ongoing randomized clinical trial [23,24]. Sim-omitted 3D treatments can carry out dosimetrically robust plans, but the implementation of sim-omitted IMRT or VMAT plans would be very challenging. The inherent setup differences between diagnostic scanners and radiotherapy machines can result in significant anatomical changes, potentially compromising target coverage and the sparing of OARs. Similarly, significant anatomical changes due to disease progression can also lead to compromised treatment outcomes.

Such complex sim-omitted treatments are often managed on ART platforms [25,26,27]. ART systems are capable of performing online re-optimization of treatment plans to account for anatomical changes and variations in patient setup positions [28,29,30,31]. Initially, a diagnostic image is used to create a pre-plan, and then the patient follows an ART workflow, which addresses plan degradation due to anatomical, setup, and CT number changes. Often, the adapted plans are able to meet all planning objectives and are deemed safe for delivery. This approach can significantly reduce patients’ wait times and enable earlier treatment starts [32,33]. Sim-omitted ART may reduce patients’ wait times, but it does not decrease the overall workload or time required compared to a conventional ART workflow [34,35]. In sim-omitted ART, the treatment planning process begins after the acquisition of diagnostic images, rather than starting the same process after CT simulation in a traditional workflow. Thus, the entire process still requires one to two weeks to complete contouring, planning, and QA. The primary distinction is that the workflow is initiated prior to the patient’s radiation oncology visit, allowing treatment to be delivered on the same day as the patient consultation. This approach remains challenging for urgent patients or situations requiring a quick turnaround.

As ART can account for anatomical changes during online adaptation, we extend this concept to inter-patient ART by creating a pool of library patients. These library patients are selected from previously treated cases and have undergone full contouring, ART pre-planning, QA, and initial physics checks. When a new patient is introduced, the pre-plan from a matching library case is exported to the adaptive planning system and treatment machines. A personalized online adaptive plan is then generated based on the new patient’s cone beam computed tomography (CBCT), inherently accounting for any anatomical differences compared to the library patient. Given the potential differences in target sizes and locations and OAR anatomies between two patients, developing a robust planning strategy is critical for the success of the inter-patient ART approach. The re-optimized plan is expected to achieve the intended dose distribution and planning objectives. As the process is simplified to DICOM transfer, total planning time can reduce from one to two weeks to just one to two hours. This reduction in time, while still enabling patients to receive complex IMRT or VMAT treatment, is particularly beneficial for urgent patients, such as those with spine metastasis who experience severe pain [9].

## 2. Materials and Methods

### 2.1. Proposed Workflow

**Figure 1:**
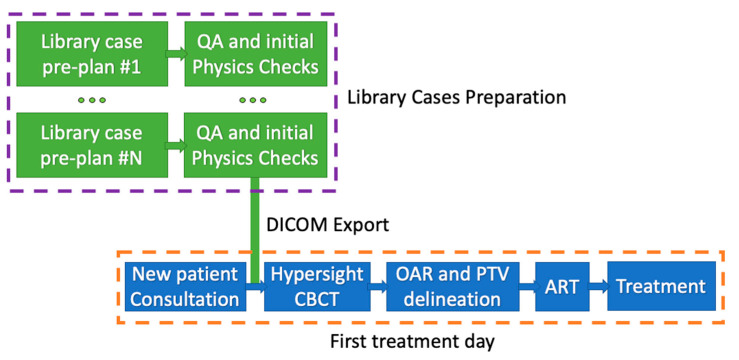
The proposed workflow. The purple box illustrates the pre-plans of library patients. This preparation work shall be completed prior to initiating the sim-omitted workflow. The DICOM export to the adaptive planning system can be completed either on or before the day of the patient’s first visit. The workflow for the first treatment day is shown in the orange box. New patients will bypass the traditional CT simulation and proceed directly with Hypersight CBCT, followed by the ART workflow to complete the treatment.

An in silico feasibility study (under institutional IRB approval) was conducted to demonstrate and evaluate the feasibility of the newly proposed workflow utilizing the Ethos Emulator 2.0 treatment planning system and ART emulator (Varian Medical System, CA) [36,37,38]. Utilizing Ethos 2.0 and Hypersight CBCT [39,40,41,42,43], direct online dose calculations on CBCT eliminate the uncertainty associated with deformable registration between two different patients caused by the synthetic CT approach in Ethos 1.0. Given the novel nature of the workflow, testing in a virtual environment was deemed a prudent approach to assessing its viability. The details of the proposed workflow are shown in Figure 1. Simulation CT scans from previously treated patients were used to construct a patient library. The library patients’ simulations CTs were contoured and pre-planned as if they were real adaptive patients, as shown in the purple dash box in Figure 1. All these plans must perform patient-specific QA and initial physics chart checks for actual clinical implementation. As a feasibility study without involving treatment delivery to actual patients, there were no patient safety concerns in this phase. To prioritize the efficiency of our study, we did not perform QAs or initial physics chart checks at this stage. This work shall be completed as part of preparation work ahead of initiating the proposed workflow. The orange dash box in Figure 1 shows the workflow of the patient’s first visit. The library patient’s simulation CT and pre-plan are imported into the Ethos planning system either before or on the day of patient treatment. Then, the regular ART workflow is followed. In our feasibility study, the new testing patient’s Hypersight CBCT was imported to the ART emulator as a sim-omitted case for online ART purposes. The ART workflow of the Ethos 2.0 system was then followed. It includes OAR and PTV contouring, ART plan generation, and finally, pseudo-treatment delivery.

### 2.2. Spine SAbR Patient Planning

The prescription of this study was 14Gy in a single fraction to the entire vertebral body, with a simultaneous integrated boost to 20Gy. The most critical OAR was the spinal cord. Its planning goals were a maximum dose of less than 14Gy and a V10Gy of less than 0.35cc. Other OARs, such as heart, lungs, great vessels, esophagus, kidneys, and others, were contoured if they were in proximity to the treatment site. Their clinical significance was deemed lower due to either their relatively greater distance from the targets or their higher dose tolerance. Their planning objectives are listed in Table 1. 

### 2.3. Library Cases and ART Strategy Development

The library was designed and pre-planned for either T or L spine SAbR treatment. In this study, five library patients’ simulation CTs were designated for the T spine site, and another four simulation CTs for the L spine site. Each pre-plan used the default setup of 12 fields in the planning system with evenly distributed gantry angles and a 10-degree collimator angle. We selected another 10 different patients, as they were ‘new patients’, to test the feasibility of the proposed workflow. They had previously been treated in our institution with Hypersight CBCTs. For each test patient, a library patient was selected based on similar anatomical features, primarily focusing on the PTV volumes and their proximity to the spinal cord.

Given that the scheduled plan from one library patient is unlikely to be suitable for delivery to a new patient, robustness of the planning template is the key priority of the proposed workflow to ensure the success of ART plans. A robust planning template would allow the generation of an adaptive plan that not only ensures adequate PTV coverage but, more importantly, spares the spinal cord and other OARs. One commonly used planning algorithm is the ranking system [44,45]. We tested multiple planning templates in this planning system and identified the following ranking template, which was robust and effective for all our spine SAbR cases, as shown in Table 1. Several planning structures were created. The PTV20Gy-Cord3 mm structure was created using PTV20Gy minus 3 mm expansion of the cord. The plan was normalized to 95.1% of the 20Gy coverage to PTV20Gy-Cord3mm. The PTV14Gy-Cord2 mm structure was created using PTV14Gy minus 2 mm expansion of the cord. Additionally, two ring structures were created to achieve conformal dose distribution: one 3 mm outside PTV20Gy, and the other 3 mm outside PTV14Gy. The top two rankings are the two spinal cord objectives in P1. PTV coverage objectives, followed by other OARs and ring structures, are in P2 and P3. The same treatment planning template was applied consistently across all spine SAbR cases.

### 2.4. Validation of Simulation-Omitted Spine SAbR

**Figure 2:**
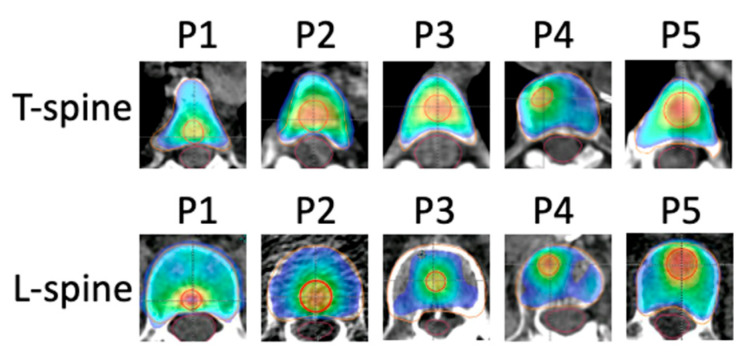
The PTV20Gy is in red and PTV14Gy is in orange. The PTV20Gy volumes and locations of all 10 patients (T and L spines patients #1–5) are shown in the figure.

The 14Gy and 20Gy PTVs of the 10 test patients are shown in Figure 2. To test the robustness of the template, we designed various PTV20Gy volumes at different proximities to the spinal cord. This design was intended to represent a diverse cohort of clinical patients. In our study, we prioritized achieving planning goals for the spinal cord, aiming for 95% PTV coverage, with 90% coverage as an alternative acceptance criterion. The robustness of the planning template was evaluated as the ability to meet both PTV coverage and OAR dose constraints in all test patients.

## 3. Results

**Figure 3:**
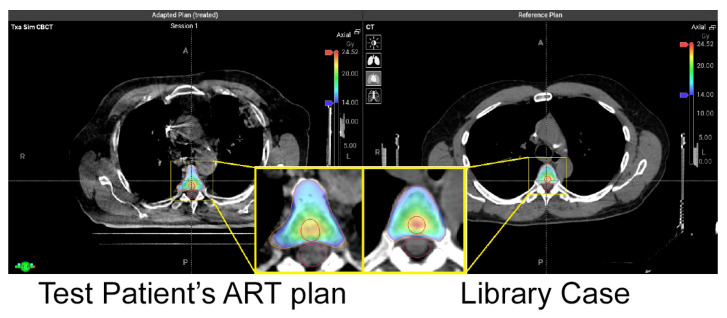
The adaptive plan of T spine patient #1 with Hypersight CBCT is shown on the left. The pre-plan from library patient #1 with simulation CT is shown on the right. The orange contour is 14 Gy PTV, and the red contour is 20Gy PTV.

Figure 3 shows an example of the library pre-plan and the corresponding Hypersight CBCT ART plan. The adaptive plan demonstrates great coverage of the targets despite the close proximity of PTV20Gy to the spinal cord. Table 2 shows five T spine patients’ key dosimetric metrics of PTV coverages and cord doses. Table 3 shows five L spine cases with the same dosimetric metrics. All adaptive PTV20Gy and PTV14Gy had dose coverage of at least above 90%. Six out of ten patients were able to reach 95% coverage of PTV20Gy. These cases primarily involved PTVs located further from the spinal cord. No violations of cord dose constraints were observed in any of the 10 ART patients. All ten test patients met PTV coverage goals and spinal cord dose constraints. The average PTV20Gy coverage was 93.9% (90.2–95.2%). Due to the cord dose constraint of V10Gy < 0.35cc, three test patients achieved 95% coverage of PTV14Gy, while all ten patients met the 90% coverage threshold. The average PTV14Gy coverage was 93.3% (90.4–98.1%).

In our study, each test patient was paired with a library patient with similar PTV volumes and proximities to the spinal cord. The online adaptive plans and pre-plans achieved similar quality. The average difference in the coverage of PTV20Gy and PTV14Gy is 1.05% (0.1–3.4%) and 1.76% (0.2–6.8%), respectively. The average difference in spinal cord dosimetry is 0.67Gy (0.09–2.5Gy) for maximum dose and 0.09cc (0–0.61cc) for V10Gy. The similarity indicates that the ART plans and pre-plans are expected to achieve similar plan quality despite differences in patients’ overall anatomy (as shown in Figure 3) and the inter-patient ART regimen. This proves the robustness of the planning template across different patients. Furthermore, when the 20Gy targets are far away from the cord, the dosimetric metrics are observed to have less variability. When they are close to the cord, larger differences in coverage and OAR doses are observed between the two plans.

## 4. Discussion

In our study, we demonstrated that our proposed sim-omitted spine SAbR workflow and inter-patients ART are feasible, providing a robust planning template that can account for anatomical differences among patients. This approach omits the CT sim, planning, QA, and initial physics chart checks. Instead, it utilizes readily available library plans associated with robust planning templates to ensure PTV coverage and OAR sparing. Therefore, it can reduce the patient’s wait time for the complex inverse planning workflow. It reduces the wait time from weeks to hours. Such a fast approach still yields a high-quality plan for treatment with great reproducibility. This approach is particularly beneficial for urgent treatments and patients with severe pain. These patients can receive IMRT-style SAbR treatments, which offer more durable disease control than traditional sim-omitted 3D treatments. In the future, we plan to investigate multi-site spine SAbR and extend this sim-omitted workflow to other sites, such as the peripheral lung, prostate, and rectum. Moreover, our approach opens the potential for treating newly developed metastases on the same day they are discovered. These metastases may not be visible on diagnostic CT, or simulation CT, but could develop during conventional or PULSAR treatment [46,47]. These new metastases can be identified on CBCT during treatment. Typically, patients have to undergo another CT simulation for a new plan. With our approach, these metastases can potentially be treated on the same day after the patient’s scheduled treatment, eliminating the need for an additional CT simulation, complex procedures, or multiple hospital visits.

We initially built our patient library by creating new plans using many simulation CT scans of previously treated patients. However, we demonstrated that a robust planning template can consistently generate ART plans, allowing a single library plan to be sufficient for treating future patients. Ethos 2.0 and Hypersight CBCT directly utilize CBCT for dose calculation. The Hounsfield units (HU) of Hypersight CBCT have been shown to be accurate across various sizes of phantoms, which is sufficient for ART planning purposes [48]. The potential uncertainties arising from differences in size between library patients and new patients are expected to be minimal. This approach could potentially eliminate the need to build a large pool of library plans for a single treatment site, thereby shortening the preparation phase of building the library and reducing the total workload for institutions aiming to implement sim-omitted treatment programs. For certain cases, such as treatments involving the posterior vertebral arch, institutions may develop separate planning templates to achieve optimized dosimetric outcomes. We also encourage institutions to develop and share their robust planning templates, leveraging the features available in the planning system to collectively improve the outcome for patients. To accumulate a library planning pool, a prospective approach involves using simulation CT scans from previously treated patients to re-plan and conduct QA for each spine or other treatment sites. Alternatively, a retrospective approach can be employed, in which existing patients for each site are accumulated over time and used to treat similar cases moving forward. While the latter approach may take longer to build the library, it requires less total planning and QA effort. Once a library pool and robust planning templates are established, the overall planning workflow can be simplified to DICOM transfers, as these library plans have been validated through QA and initial physics chart checks.

While our study demonstrates the feasibility and promise of sim-omitted spine SAbR, there are practical considerations that must be addressed for broader implementation. Importantly, we also offer strategies to mitigate these challenges. First, our feasibility study utilized the default 12-field beam arrangement. We anticipate that plan quality and dosimetric metrics could be further improved by increasing the number of fields, along with optimized gantry and collimator angles. Second, our approach requires the patient’s full anatomy to be captured in a single CBCT. For patients with larger body habitus, additional consideration during the pre-planning phase may be needed to avoid beam paths that could be attenuated or compromised. Institutions may consider modifying beam arrangements or acquisition protocols to ensure full coverage and reduce dose calculation uncertainties. Third, patients with prostheses, titanium implants, or complex spinal anatomies (e.g., scoliosis) may require extra care in both preplanning and online ART to ensure robustness and reproducibility. Our experience suggests that these cases can still be managed effectively with tailored workflows. Fourth, we observed that Ethos 2.0, which uses artificial intelligence (AI)-generated body contours, may inconsistently include or exclude immobilization devices, such as body frames—unlike Ethos 1.0, which uses deformed planning contours. As the AI contouring mechanism remains a black box, this variability should be considered when designing sim-omitted workflows. At our institution, we address this issue by developing a radiation-transparent version of our body frame. Alternatively, for appropriate patients, a frameless CBCT-guided workflow may be considered to simplify implementation.

## 5. Conclusions

This study suggests that inter-patient ART is a feasible and promising approach for spine SAbR, offering a simulation- and planning-free workflow that eliminates the need for CT simulation, planning, pre-treatment QA, and initial physics chart checks. By using a library of pre-plans from previously treated patients and developing robust planning strategies, this approach can account for the anatomical differences between patients. Our study included cases with varying PTV volumes, shapes, and proximity to OARs. Initial findings indicate that a robust planning template can be developed, though further validation across a larger cohort of patient population is still necessary. The proposed workflow has the potential to significantly reduce preparation time—from weeks to hours—while maintaining high-quality, reproducible treatment plans. This method may enable patients to start their treatments quickly, which is particularly beneficial for urgent cases, such as patients with spine metastases experiencing severe pain, potentially allowing them to receive IMRT-style SAbR treatments that offer more durable disease control compared to traditional 3D treatments.

Looking forward, the proposed workflow can be extended to multi-site spine SAbR and other treatment sites, such as the peripheral lung, prostate, and rectum. Additionally, this method may enable the treatment of newly discovered lesions during the same treatment session, bypassing another round of CT simulation and allowing immediate treatment upon discovery. This innovative approach has the potential to enhance clinical efficiency and improve patient care, particularly for patients requiring urgent interventions.

## Figures and Tables

**Table 1:** Treatment planning goals and ranking of the planning template.

Ranking ^a^	Structure	Primary Goal	Alternative Goal
P1 Most Important	Spinal Cord	V10Gy ≤ 0.35cc	
P1	Spinal Cord	D0.03cc ≤ 14Gy	
P2 Very Important	* PTV20Gy-Cord3mm ^b^	D10% ≥ 110%	
P2	* PTV14Gy-Cord2mm ^b^	V100% ≥ 96%	
P2	* PTV20Gy-Cord3mm ^b^	V100% ≥ 95.1%	
P2	PTV20Gy	V100% ≥ 95.1%	≥90%
P2	PTV14Gy	V100% ≥ 95.1%	≥90%
P2	* Ring14Gy3mm ^c^	Dmax ≤ 12Gy	
P2	* Ring20Gy3mm ^c^	Dmax ≤ 15Gy	≤20Gy
P2	Heart	V16Gy ≤ 15cc	
P2	Heart	D0.03cc ≤ 22Gy	
P2	Small Bowel	D0.03cc ≤ 20Gy	
P2	Esophagus	V20Gy ≥ 5cc	
P2	Esophagus	D0.03cc ≤ 24Gy	
P2	Kidney left	D33.4% ≤ 9.5Gy	
P2	Kidney left	V14Gy ≤ 15cc	
P2	Kidney right	D33.4% ≤ 9.5Gy	
P2	Kidney right	V14Gy ≤ 15cc	
P2	Both Kidneys	D33.4% ≤ 9.5Gy	
P2	Small Bowel	V7.6Gy ≤ 30cc	
P2	Skin	D0.03cc ≤ 27.5Gy	
P2	Skin	V25.5Gy ≤ 10cc	
P2	Both Lungs	D33.3% ≤ 7.2Gy	
P3 Important	Both Lungs	D950cc ≤ 7.2Gy	
P3	Both Lungs	D1500cc ≤ 7.2Gy	
P3	Both Lungs	V8Gy ≤ 37%	

Note: * means the structure is planning structure, not actual PTV or OAR. ^a^ PX means priority X in the Ethos planning system; ^b^ PTVXXGy-CordXmm means XXGy PTV subtracts spinal cord with X mm expansion; ^c^ RingXXGy3mm is a ring structure created 3 mm outside the PTVXXGy structure.

**Table 2:** T spine patients # 1–5 (P1–P5) dosimetric metrics.

	P1		P2		P3		P4		P5	
	Library Pre-Plan	Test ART	Library Pre-Plan	Test ART	Library Pre-Plan	Test ART	Library Pre-Plan	Test ART	Library Pre-Plan	Test ART
PTV20GY(%)	89.5	90.2	89.1	92.2	95.5	95.1	95.0	95.2	95.0	95.1
PTV14GY(%)	96.7	92.9	95.2	95.4	92.8	92.0	93.9	92.0	93.2	93.0
CORD MAX (GY)	12.2	12.0	11.7	12.3	10.5	11.2	10.8	10.7	10.5	11.1
CORD V10GY (CC)	0.20	0.15	0.14	0.14	0.06	0.08	0.09	0.08	0.08	0.09

**Table 3:** L spine patients # 1–5 (P1–P5) dosimetric metrics.

	P1		P2		P3		P4		P5	
	Library Pre-Plan	Test ART	Library Pre-Plan	Test ART	Library Pre-Plan	Test ART	Library Pre-Plan	Test ART	Library Pre-Plan	Test ART
PTV20GY(%)	87.9	91.3	92.6	94.9	95.0	95.1	95.0	95.1	95.3	95.1
PTV14GY(%)	97.3	98.1	97.2	90.4	92.8	91.3	91.9	92.6	94.3	95.2
CORD MAX (GY)	13.7	13.3	13.5	11.0	10.4	9.4	10.2	10.6	10.4	10.2
CORD V10GY (CC)	0.21	0.24	0.77	0.16	0.09	0.00	0.04	0.08	0.06	0.05

## Data Availability

Due to HIPAA and PHI concerns, we will not provide patient data publicly. However, we may provide patient data in an appropriate format that complies with current laws and ethical guidelines upon request.
